# Skeletally immature patients undergoing over‐the‐top anterior cruciate ligament (ACL) reconstruction using hamstring autograft with preserved tibial insertion and lateral extraarticular tenodesis (LET) exhibit good early graft MRI signal similar to adult patients

**DOI:** 10.1002/jeo2.70590

**Published:** 2026-04-16

**Authors:** Yuta Nakanishi, Luca Ambrosini, Emanuele Altovino, Claudio Rossi, Emre Anil Özbek, Stefano Zaffagnini, Alberto Grassi

**Affiliations:** ^1^ Department of Orthopaedic Surgery Kobe University Graduate School of Medicine Kobe Japan; ^2^ Alma Mater Studiorum Università di Bologna Bologna Italy; ^3^ II Clinica Ortopedica e Traumatologica IRCCS Istituto Ortopedico Rizzoli Bologna Italy; ^4^ Department of Orthopaedic Surgery Ankara University Ankara Turkey; ^5^ Dipartimento di Scienze Biomediche e Neuromotorie (DIBINEM) Università di Bologna Bologna Italy

**Keywords:** ACL reconstruction, graft maturation, lateral extraarticular tenodesis, physeal sparing, skeletally immature

## Abstract

**Purpose:**

To evaluate short‐term anterior cruciate ligament (ACL) graft maturity in skeletally immature patients undergoing ACL reconstruction (ACLR) with physeal‐sparing over‐the‐top (OTT) technique using hamstring tendon (HT) autograft with preserved tibial insertion, and to compare the results with adult patients operated using an OTT technique with similar features.

**Methods:**

Skeletally immature patients who underwent primary ACLR with OTT between February 2022 and January 2025 with post‐operative Magnetic Resonance Imaging (MRI) performed between 10 weeks and 6 months were retrospectively reviewed. Graft maturation was evaluated via the Howell grading system and ACL signal/noise quotient (SNQ) on MRI. Additionally, graft continuity, tunnel widening, fluid collection within the graft, and bone oedema of the tibial tunnel wall were assessed. Skeletally immature patients were propensity‐matched at a 1:1 ratio to adult patients, and comparisons were performed.

**Results:**

A total of 22 skeletally immature patients (average skeletal age 12.9 ± 2.3 years) out of 79 patients were included. MRI assessment of graft maturity was performed at an average of 4.0 ± 1.3 months postoperatively. All patients presented graft continuity, with Grade I or II Howell grade in 86% of cases. For the comparative analysis, a subset of 10 skeletally immature patients (those with a tibial tunnel) was matched with 10 adult patients (90% males, mean age 25.9 ± 10.0 years) who underwent MRI 4.0 ± 1.2 and 18.0 ± 2.1 months after surgery. No significant differences were reported for all individual items, such as the Howell graft score, SNQ, and tunnel features, between skeletally immature and adult patients at the 4‐month assessment (*p* > 0.05).

**Conclusion:**

ACLR with OTT technique via HT autograft with preserved tibial insertion may provide satisfactory ligamentization in skeletally immature patients. Graft maturity was comparable to that of the adult population. These data suggest that graft maturation using this specific surgical approach is satisfactory in skeletally immature patients and is comparable to adults.

**Level of Evidence:**

Level IV, retrospective study.

## Graphical abstract

Over‐the‐top ACL reconstruction with preserved hamstring insertion and LET in skeletally immature patients resulted in early graft maturity, comparable to adults at either 4 and 18 months post‐surgery.

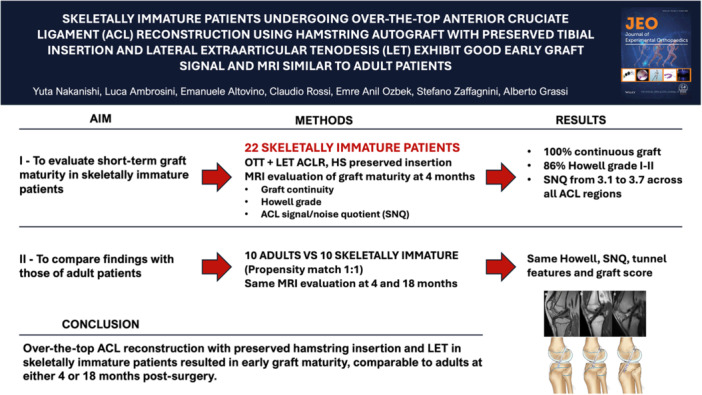

**Abbreviations**
ACL: anterior cruciate ligament
ACLR: anterior cruciate ligament reconstruction
BMI: body mass index
HT: hamstring tendon
LET: lateral extra‐articular tenodesis
LMORT: lateral meniscus oblique radial tear
MRI: magnetic resonance imaging
OTT: over‐the‐top technique
ROI: regions of interest
SNQ: signal noise quotient
STIR: Short Tau Inversion Recovery

## INTRODUCTION

Despite a greater biological healing potential, paediatric patients have been reported to exhibit higher anterior cruciate ligament reconstruction (ACLR) failure rates and longer graft ligamentization times compared to adults [3, 9, 16, 24]. Ligamentization, which reflects graft biomechanics and vascularisation, is considered a determinant of ultimate load to failure, tensile strength, and stiffness [26]. A better understanding of this process may refine return‐to‐sport decisions, as premature activity has been identified as a major risk factor for anterior cruciate ligament (ACL) graft failure in children [2, 6].

While delayed ligamentization in paediatric patients has been consistently reported, the timeframe remains variable, ranging from 6 months to 3 years [1, 5, 8, 20]. Graft selection has been suggested to influence this delayed maturation. Hamstring tendon (HT) autografts have been associated with prolonged ligamentization in paediatric patients [20], possibly due to age‐related physiological knee laxity [1]. On the other hand, lateral extra‐articular tenodesis (LET) has been shown to enhance graft maturity. Several studies comparing isolated ACLR with ACLR + LET over 6 months to 2 years found that the latter group exhibited better magnetic resonance imaging (MRI) signal intensity of the ACL graft, irrespective of whether HT or quadriceps tendon grafts were used [5, 22, 28]. Furthermore, preserving the tibial insertion of HT autografts has been associated with improved graft maturity [11], knee stability and lower re‐tear rates [25], suggesting a potential strategy to optimise outcomes in paediatric ACLR.

The primary aim of the present study was to evaluate short‐term graft maturity in skeletally immature patients. It was hypothesised that satisfactory graft maturation could be achieved in the short term. The secondary aim was to compare findings with those of adult patients undergoing ACLR with the Over‐The‐Top technique (OTT) via HT autograft with preserved tibial insertion. It was further hypothesised that results will be comparable to those seen in adult patients.

## METHODS

Approval of this study was obtained from the local Ethical Committee (CE‐AVEC 380/2019/Oss/IOR, protocol number 0006881). A retrospective review was conducted on all primary ACLR in skeletally immature patients performed at the IRCCS Rizzoli Orthopedic Institute between February 2022 and January 2025 by a single surgeon (A.G.). Patients were considered “skeletally immature” if either the tibial or femoral physis was open on their knee MRI based on the Politzer et al. shorthand of the Pennock Atlas [21].

### Patient selection

Inclusion criteria for the skeletally immature cohort were: primary ACL using supra‐, extra‐ or trans‐physeal OTT technique with LET, skeletally immature status, and a post‐operative MRI performed between 10 weeks and 6 months after surgery for any reason (rehabilitation progression, return to play, meniscal repair assessment, knee pain or new traumas). Exclusion criteria comprised multi‐ligament injuries, revision ACL procedures, associated cartilage tears, absence of the required post‐operative MRI, closed physes, body mass index (BMI) > 30, and poor patient compliance.

A control group of skeletally mature adult patients who underwent ACL reconstruction with the over‐the‐top technique was created. A 1:1 matching was then performed between this adult pool and the cohort of skeletally immature patients. To ensure a valid comparison of tibial tunnel features on MRI among skeletally immature patients, only who received a trans‐physeal or supra‐physeal technique (with a tibial tunnel) were eligible for matching; those receiving an extra‐physeal technique were excluded. Regarding skeletally mature adult patients, inclusion criteria were primary ACLR using trans‐physeal OTT technique with LET, and the completion of two protocol‐defined post‐operative MRIs (one between 10 weeks to 6 months and another after 12 months). Exclusion criteria included multi‐ligament injuries, revision procedures, associated cartilage tears, absence of the required MRI, BMI > 30, and poor compliance. Adult patients were extracted from a prospective database of 30 patients included in a randomised controlled study aimed at evaluating the outcome of different techniques for ACL reconstruction with navigation and that included post‐operative MRI as a part of the study protocol [4]. Matching was performed based on a propensity score including surgical technique, sex and meniscal lesions.

### Surgical technique

**Figure 1:**
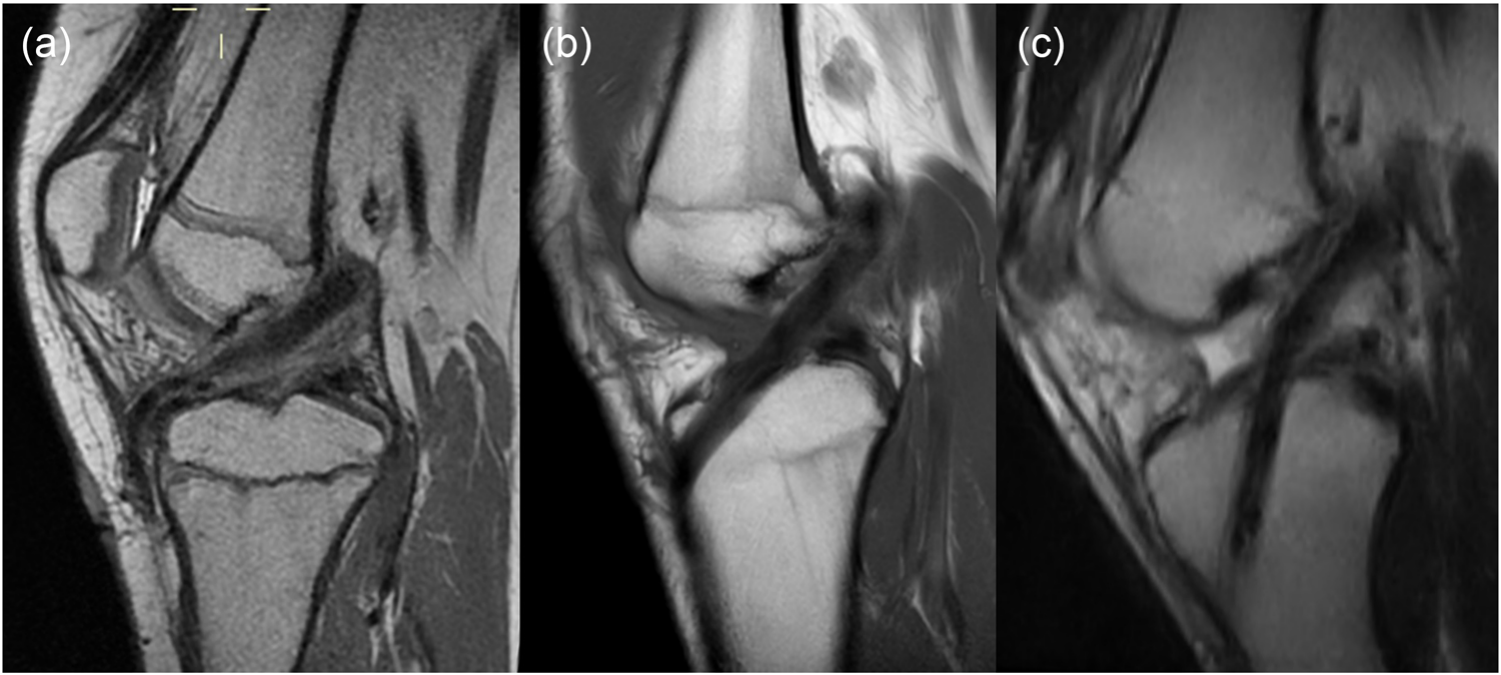
Magnetic resonance imaging (MRI) illustrating the three techniques for tibial graft passage. Single‐stranded gracilis and semitendinosus tendons passed (a) over the tibial epiphysis and beneath the intermeniscal ligament, (b) through a supra‐physeal tunnel, and (c) through a trans‐physeal tunnel.

**Figure 2:**
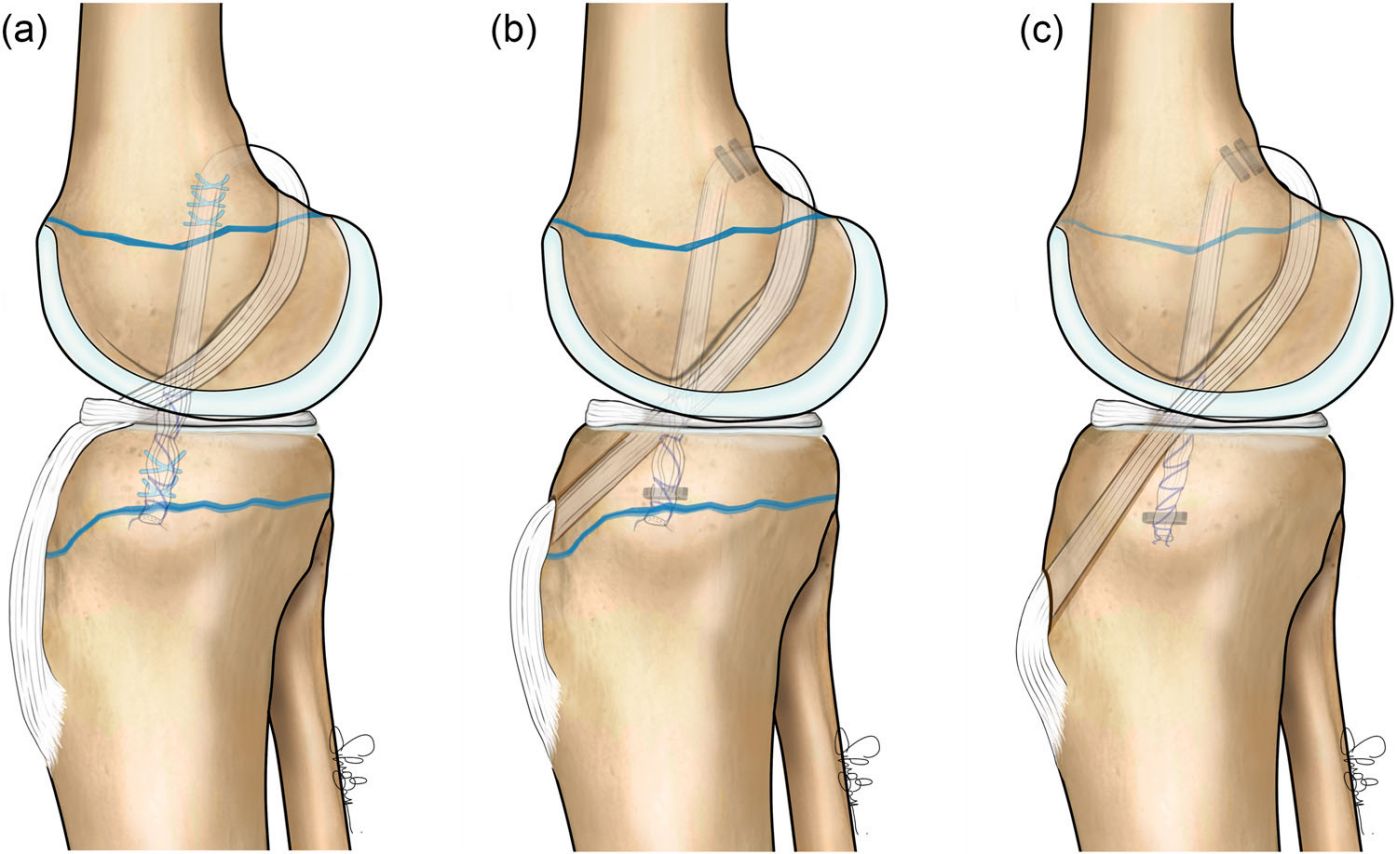
Schematic illustration of the three techniques for tibial graft passage. Single‐stranded gracilis and semitendinosus tendons passed (a) over the tibial epiphysis and beneath the intermeniscal ligament, (b) through a supra‐physeal tunnel, and (c) through a trans‐physeal tunnel.

All patients were operated on with an age‐specific variation of the OTT and lateral tenodesis technique described by Marcacci et al. [17]. All the variants had in common the use of the OTT approach for femoral fixation, thus resulting in no femoral tunnel, the systematic execution of a LET, and the preservation of the hamstrings' tibial attachment [18]. The execution of the tibial tunnel was determined based on each patient's tibial bone age according to previous literature [10]. In cases with open tibial physis in males < 12 years and females < 10 years of skeletal age, no tunnels were drilled; instead, the single‐stranded gracilis and semitendinosus tendons were passed over the tibial epiphysis and beneath the intermeniscal ligament (Figures 1a and 2a). For open tibial physis in males > 12 years and females >10 years of skeletal age, a supra‐physeal tunnel was drilled, and single‐strand gracilis and semitendinosus tendons were passed through it (Figures 1b and 2b). In cases with closed tibial physis, a trans‐physeal tunnel was drilled, and single‐stranded gracilis and semitendinosus tendons were passed through it (Figures 1c and 2c). Femoral fixation was supra‐physeal for all patients. If skeletal age was > 12 years in males and > 10 years in females, metal staples were used. Otherwise, fixation was achieved with trans‐osseous sutures.

### Post‐operative graft maturity MRI assessment

All MRIs were performed between 10 weeks and 6 months after surgery as part of the study protocol. Senior author (A.G.) evaluated all MRIs using the Radiant Dicom Viewer (Medixant, Poland). Graft evaluation was performed according to the scale by Grassi et al. [11] Briefly, the evaluation considered graft continuity, the overall graft appearance according to the Howell Grading system, the presence of fluid collection within the graft and the graft signal using the signal/noise quotient (SNQ). Moreover, the features of the tibial tunnel (when present) were evaluated considering the signal of the graft inside the tunnel, the presence of fluid collection in the graft, bone oedema surrounding the tibial tunnel, and tunnel widening. Each item is described as follows.

### Graft continuity

Defined as the clear presence of the graft without interruptions in all sagittal slices of the intercondylar notch volume. If the graft was continuous, ‘1 point’ was assigned; otherwise ‘0 points’ were assigned.

### Howell grade

Grade I was assigned to a graft with a homogeneous low‐intensity signal which is not different from the signal of the native quadriceps tendon; Grade II was assigned to a graft where < 50% of the fibres have acquired a higher intensity signal; Grade III was assigned to a graft where ≥ 50% of the fibres have acquired a higher signal intensity; Grade IV was assigned to a graft which signal is diffusely increased without normal‐appearing fibres. ‘2 points’ were assigned in the case of a Grade I graft, ‘1 point’ in the case of a Grade II graft and ‘0 points’ in the case of a Grade III or IV graft [12, 13].

### ACL signal/noise quotient

**Figure 3:**
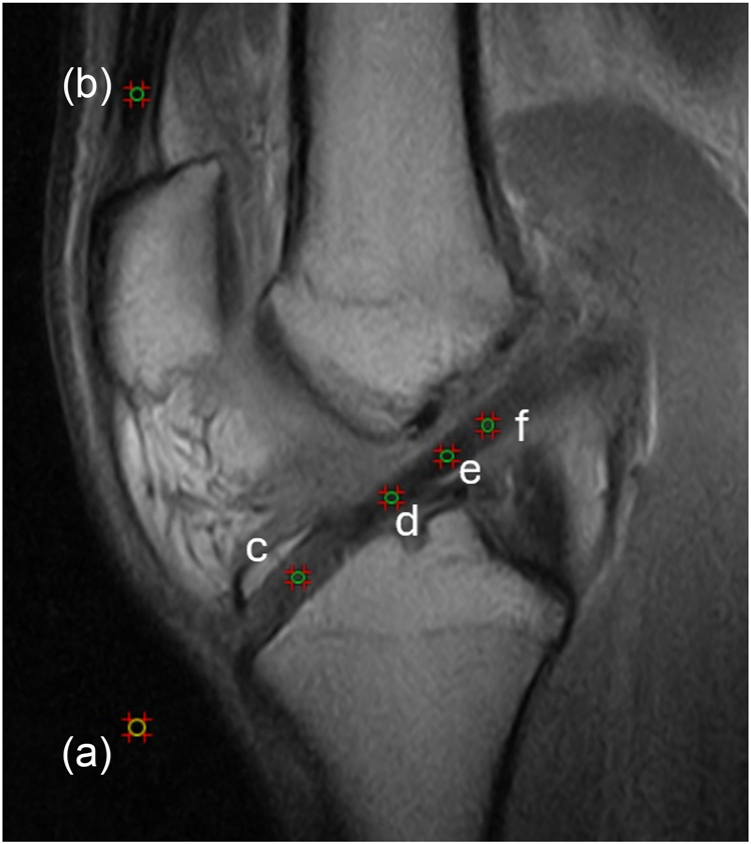
Designated regions of interest (ROI) were (a) background signal (2 cm anterior to the patellar tendon), (b) midportion of the quadriceps tendon, (c) portion of the graft inside the tunnel, (d) distal, (e) intermediate and (f) proximal intra‐articular portion of the graft.

The signal intensity was calculated using a 2 mm × 2 mm circle in precise regions of interest (ROI) (Figure 3), which were the proximal, intermediate, and distal intra‐articular portions of the graft, portion of the graft inside the tunnel, the mid‐portion of the quadriceps tendon, and the background signal (2 cm anterior to the patellar tendon). To obtain the normalised signal, SNQ was calculated for each ROI using the formula SNQ = (signal of the ROI − signal of the quadriceps tendon)/background signal. ‘2 points’ were assigned if at least two ROI had SNQ < 1, ‘1 point’ if only one area had SNQ < 1, otherwise ‘0 points’ were assigned [14].

### Fluid collection within the graft

**Figure 4:**
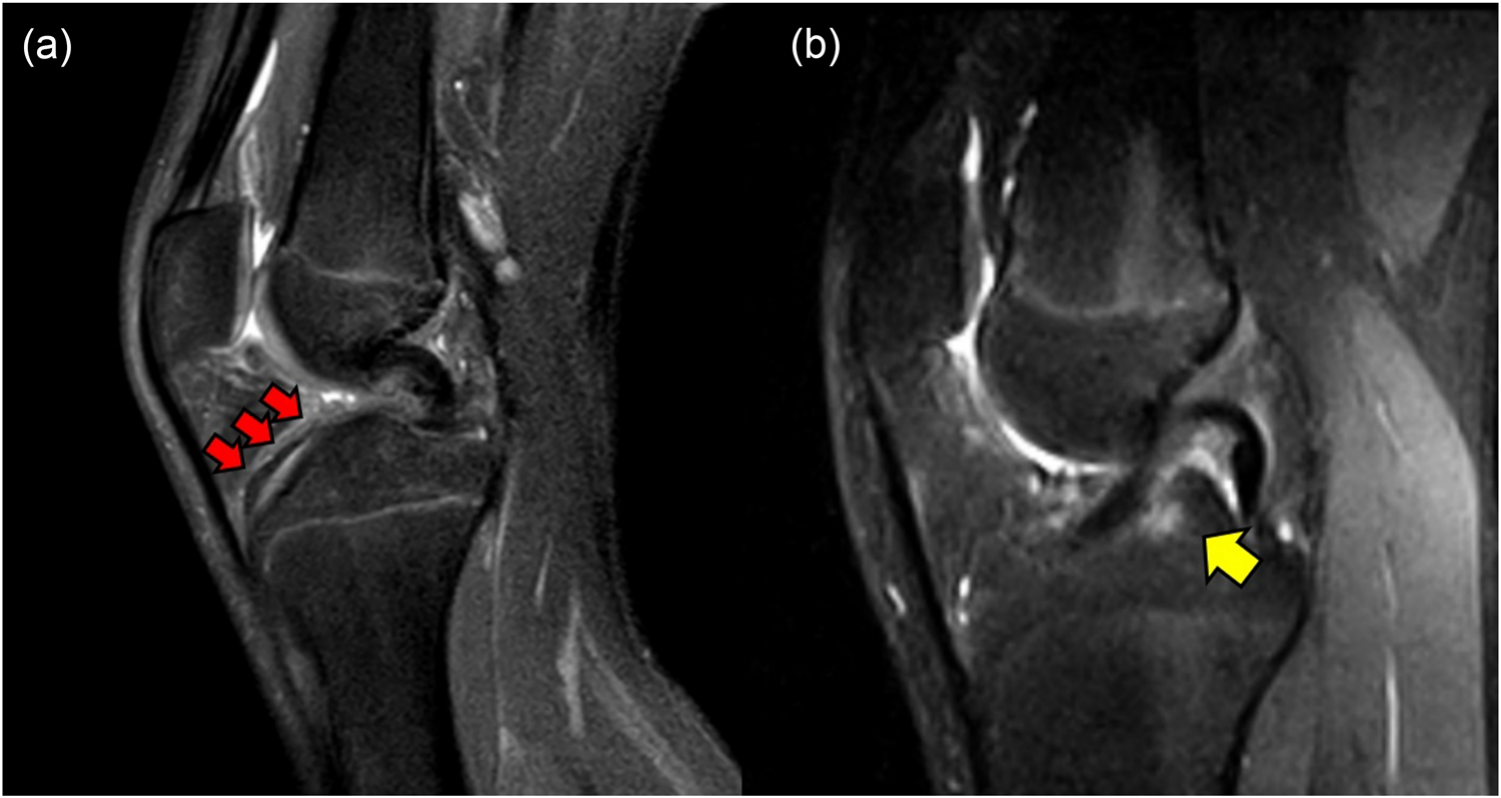
Presence of a hyperintense signal band between the fibres alongside a low‐intensity signal within the graft (a) and a hyperintense signal area in the cancellous bone around the graft (b) at short tau inversion recovery (STIR) magnetic resonance imaging scans.

The oedematous fluid within the graft was evaluated using the short tau inversion recovery (STIR) MRI scans and identified as the presence of a hyperintense signal band or cyst between the fibres alongside a low‐intensity signal; ‘1 point’ was assigned in cases of fluid collection absence, while ‘0 points’ were given if fluid collection was present within the graft (Figure 4a).

### Bone oedema of the tibial tunnel walls

The oedematous condition of the tunnel surroundings evaluated on the STIR MRI scans were defined as the presence of a hyperintense signal area in the cancellous bone around the graft; ‘1 point’ was assigned in the absence of oedema, and otherwise, ‘0 points’ were assigned (Figure 4b).

### Signal/noise quotient of the graft inside the tibial tunnel

Similar to the intra‐articular portion of the graft, the signal of the graft inside the tibial tunnel was calculated using a 2 mm × 2 mm circle in a precise ROI, which was at least 1 cm distal from the tibial articular surface (Figure 3). The same formula was used to obtain the SNQ of the intra‐tunnel portion of the graft; in the case of SNQ < 1, ‘1 point’ was assigned, otherwise ‘0 points’ were assigned.

### Tunnel widening

Defined as the difference between the tunnel width calculated on the MRI measured as the maximum distance between the anterior tibial cortex and the posterior tunnel wall on sagittal slice and the initial drilled diameter recorded in the surgical reports. If the widening was < 1 mm, ‘1 point’ was assigned, otherwise ‘0 points’ were assigned.

### Overall grading

Based on the MRI evaluations and the points assigned to each parameter, a 0–6 points were assigned to assess the graft status and from 0 to 4 points were assigned for tunnel features. Therefore, an overall score of MRI graft maturation ranging from 0 to 10 was finally obtained. The reliability of the used MRI features were already determined in previous studies [11].

### Statistical analysis

An a‐priori sample size calculation was based on the total graft score, using the values from Grassi et al. [11] which compared the ligamentization of two different ACL reconstruction techniques. Considering an Alpha error (significance) of 0.05 and a Beta error (1‐power) of 0.10, a total of eight patients per group were required. A total of 10 patients per group were included to consider possible dropouts. The statistical analysis was performed using the statistical software MedCalc (version 22.023). Continuous variables were reported as mean ± standard deviation, while categorical variables were reported as a raw number and percentage of the total. Continuous variables were compared using the independent sample t‐test when comparing the two groups. Categorical variables were compared using the chi‐squared test or the 2 × 2 Fisher exact test based on the number of variables considered. Values were considered statistically significant with *p* < 0.05.

## RESULTS

### Patient characteristics

A total of 79 skeletally immature patients underwent ACL reconstruction within the considered time frame (Table 1). After applying the inclusion and exclusion criteria, 57 patients were excluded due to the absence of the required postoperative MRI. The final cohort resulted in 22 included patients, with a mean chronological age of 13.5 ± 1.9 years and a mean skeletal age of 12.9 ± 2.3 years (Table 2). The primary indications for the post‐operative MRI, performed between 10 weeks and 6 months of surgery, were as follows: to evaluate healing after bucket handle meniscus repair (*n* = 4), to assess lateral meniscus oblique radial tear (LMORT) recovery (*n* = 6), to determine readiness for rehabilitation progression (*n* = 8), and to obtain clearance for non‐contact sports (*n* = 4). Regarding surgical technique, 10 patients (46%) were operated with an extra‐physeal technique without a tibial tunnel and 12 patients (54%) with a tibial tunnel, specifically 6 (27%) with a supra‐physeal technique and 6 (27%) with a trans‐physeal technique.

**Table 1:** Demographic details.

Sex (M/F)	66 (83%)/13 (17%)
Chronological age	13.9 ± 2.2 years
Males	13.9 ± 2.3 years
Females	13.8 ± 1.5 years
Skeletal age	14.0 ± 2.3 years
Males	14.0 ± 2.5 years
Females	14.3 ± 1.2 years
Difference between bone and chronological age	0.1 ± 0.9 years
Years of remaining growth based on chronological age	1.9 ± 2.3 years
Years of remaining growth based on bone age	1.9 ± 2.2 years

**Table 2:** Demographics and MRI evaluation after ACL reconstruction.

Demographics	Values
Skeletal age at surgery (years)	12.9 ± 2.3
Chronological age at surgery (years)	13.5 ± 1.9
Sex (M/F)	19 (86%)/3 (14%)
Physeal‐sparing technique	
Extra‐physeal OTT	10 (46%)
Supra‐physeal OTT	6 (27%)
Trans‐physeal OTT	6 (27%)

MRI graft evaluation	Values
MRI assessment (months)	4.0 ± 1.3
Graft continuity	22 (100%)
Howell grade	
I	9 (41%)
II	9 (45%)
III	3 (14%)
IV	0 (0%)
Fluid collection within graft	1 (5%)
SNQ ACL (mean)	
Distal	3.1 ± 3.6
Middle	3.5 ± 3.6
Proximal	3.7 ± 3.8
SNQ ACL (value < 1)	
Distal	9 (41%)
Middle	8 (36%)
Proximal	6 (27%)
Graft Score (0–6)	4.1 ± 1.5

Abbreviations: ACL, anterior cruciate ligament; MRI, magnetic resonance imaging; OTT, over‐the‐top; SNQ, signal/noise quotient.

### MRI graft assessment of skeletally immature patients

The MRI assessment of graft maturity was performed at an average of 4.0 ± 1.3 months after surgery (range 2.5–6.0 months). All patients presented graft continuity, with Grade I and Grade II Howell grade in 86% of cases, and only one patient (5%) had fluid collection within the graft strands. The average SNQ ranged from 3.1 ± 3.6 to 3.7 ± 3.8 in the various ACL regions (Table 2); the SNQ was < 1 in 27%–41% of patients depending on the region.

### MRI graft assessment of skeletally immature patients compared with adult patients

A total of 30 adult patients underwent ACL reconstruction within the considered time frame. After applying the inclusion and exclusion criteria, 20 patients were excluded due to the absence of the required postoperative MRI.

The matching process created 10 matched pairs for comparative analysis. The final groups consisted of 10 skeletally immature patients (mean age 15.3 ± 1.0, 90% males) and 10 adult patients with closed physes (mean age 25.9 ± 10.0 years, 90% males). The baseline characteristics of these matched cohorts are detailed in Table 3.

**Table 3:** MRI evaluation of patients with open physes versus adults.

	Open physes (4 months MRI)	Adults (4 months MRI)	Adults (18 months MRI)	*p* value (open vs. adults 4 m)	*p* value (open vs. adults 18 m)
Number of patients	10	10	10		
MRI assessment (months)	4.3 ± 1.7	4.0 ± 1.2	18.0 ± 2.1		
Age at MRI (years)	15.3 ± 1.0	25.9 ± 10	27.2 ± 10		
Graft evaluation					
Graft continuity	10 (100%)	10 (100%)	10 (100%)	=1.00	=1.00
Howell grade				=0.79	=0.63
I	4 (40%)	6 (60%)	8 (80%)		
II	5 (50%)	4 (40%)	2 (20%)		
III	1 (10%)	0 (0%)	0 (0%)		
IV	0 (0%)	0 (0%)	0 (0%)		
Liquid within graft	0 (0%)	2 (20%)	1 (10%)		
SNQ ACL (mean)					
Distal	2.46 ± 2.68	2.60 ± 2.70	1.67 ± 2.17	=0.91	=0.48
Middle	2.18 ± 1.84	2.62 ± 2.36	3.10 ± 1.40	=0.65	=0.22
Proximal	2.04 ± 2.08	3.20 ± 2.58	2.48 ± 1.45	=0.28	=0.59
SNQ ACL (value < 1)					
Distal	4 (40%)	3 (30%)	5 (50%)	=1.00	=1.00
Middle	4 (40%)	3 (30%)	0 (0%)	=1.00	=0.09
Proximal	4 (40%)	1 (10%)	1 (10%)	=0.30	=0.30
Tunnel evaluation					
SNQ graft inside tunnel (mean)	0.89 ± 2.40	0.39 ± 1.46	0.15 ± 0.91	=0.58	=0.37
SNQ graft inside tunnel ( < 1)	7 (70%)	9 (90%)	9 (90%)	=0.58	=0.58
Tunnel bone oedema	2 (20%)	1 (10%)	0 (0%)	=1.00	=0.47
Liquid within tunnel	2 (20%)	0 (0%)	1 (10%)	=0.47	=1.00
Tunnel enlargement >1 mm	0 (0%)	0 (0%)	0 (0%)	=1.00	=1.00
Tunnel Score (0–4)	3.3 ± 0.7	3.6 ± 0.7	3.8 ± 0.4	=0.35	=0.07
Graft Score (0–6)	4.3 ± 1.0	3.0 ± 0.9	2.8 ± 0.6	**=0.01** *	**=0.001** *
Total Score (0–10)	7.6 ± 1.5	6.6 ± 1.3	6.6 ± 0.7	=0.13	=0.07

Abbreviations: ACL, anterior cruciate ligament; MRI, magnetic resonance imaging; SNQ, signal/noise quotient.

* *p* < 0.05.

MRI findings of skeletally immature patients (assessed at 4.3 ± 1.7 months post‐surgery) were compared to those of adult patients at two time points: mean of 4.0 ± 1.2 and 18.0 ± 2.1 months postoperatively.

No significant differences were reported for all individual items such as Howell graft score, SNQ, and tunnel features between patients with skeletally immature and adult patients at the 4 months assessment (*p* > 0.05). No significant differences were noted comparing the 4 months assessment of skeletally immature patients with the 18 months assessment of adult patients (*p* > 0.05) (Table 3). The only significant difference reported in both comparisons was a higher composite ‘graft score’ in the skeletally immature patients (*p* < 0.05). However, no significant difference for the composite “total score” was found between two groups (*p* > 0.05) (Table 3).

## DISCUSSION

The most important finding of the present study was that satisfactory graft maturation in skeletally immature patients was achieved at 4 months post‐operative follow‐up. Graft continuity was confirmed in all patients, a normal or nearly normal Howell grade (Grade I or II) was observed in 87% of cases, and the average SNQ ranged from 3.1 to 3.7 across the various ACL graft regions. Furthermore, the graft maturation total score at 4 months in the skeletally immature group was comparable to that of adults at both 4 and 18 months postoperatively.

Previous investigations have reported that ACL graft maturation in paediatric patients proceeds as a substantially slower process [3]. A study involving patients with open growth plates who underwent primary ACL reconstruction with either HT, quadriceps tendon, or iliotibial tract autografts found that ACL graft maturation continues for up to 24 months after surgery. Subsequent research showed continuous improvement in Howell grade from 6 to 24 months of follow‐up, while the SNQ score improved significantly from 6 to 12 months. However, even at 24 months, graft maturation remained suboptimal compared to the native ACL [20]. A recent study further supported that graft maturation in adolescents progresses over time but is delayed compared with adults, with Howell Grades I or II increasing from 79.5% at 6 months to 90% at 24 months, and mean SNQ decreasing from 5.2 to 3.9 over the same period [1]. Conversely, one previous study reported no difference in graft maturation on MRI across age groups; however, because skeletal maturity was not specifically assessed, the youngest cohort may not have represented truly skeletally immature individuals [23].

The surgical technique combining LET with preserved hamstring tibial insertion may have contributed to the results in this study. The positive impact of LET augmentation is consistent with previous literature across different graft types and age groups. ACLR using HT autografts combined with LET has demonstrated superior graft incorporation and significantly lower SNQ values compared to isolated ACLR at one year postoperative in adult patients [5]. Similarly, significantly lower SNQ (2.8) value have been reported for ACLR with HT autograft and anterolateral reconstruction compared to isolated procedures (4.7) [27]. These findings appear consistent across other graft types and techniques, including quadriceps tendon autografts with LET and double bundle reconstruction with anterolateral reconstruction [22, 28]. This consistent effect may be attributed to the biomechanical role of LET, which cadaveric studies have shown to reduce tensile load on the ACL by up to 80% [7, 19].

Preservation of the tibial insertion of the hamstring autograft may further promote graft maturity. Previous research demonstrated significantly superior overall graft maturation scores at 4 months post‐operatively for HT autograft with preserved tibial insertion and LET compared to those with detached tibial insertion and without lateral tenodesis [11]. However, a direct comparative study between HT autografts with and without extra‐articular tenodesis, specifically evaluating the effect of maintained tibial insertion, is warranted to confirm this hypothesis.

Interestingly, no significant differences were found between MRI evaluations at 4 months post‐operative in skeletally immature patients and 18 months in adults, suggesting an early plateau in graft maturation. This stabilisation in SNQ values is consistent with previous reports describing stable, relatively low signal intensity from 3 to 24 months post‐operatively in grafts with preserved hamstring insertion [15]. A 5‐year randomised controlled study also demonstrated that HT autografts with preserved tibial insertion exhibited significantly lower and more stable SNQ values from 6 to 24 months compared with free HT autografts [29].

This study has several limitations that should be acknowledged, including a small sample size and the absence of a control group using a standard single‐bundle technique, which may limit the generalisability of the findings. Some patients were approaching skeletal maturity with limited remaining growth, potentially influencing the applicability of the findings to younger populations with greater growth potential. Variability in MRI protocols across institutions may have introduced inconsistencies in imaging quality and interpretation. The lack of serial MRI evaluations limited the ability to assess longitudinal changes, and no correlation was made between graft signal and patient‐reported outcomes or objective laxity. Additionally, although all techniques involved preservation of the hamstring tendon insertion, variation in the over‐the‐top passage, LET fixation, and tibial tunnel positioning among cases may have introduced outcome variability.

Despite these limitations, this is the first study to the authors' knowledge to evaluate graft maturity on MRI following LET combined with hamstring insertion preservation using the OTT in skeletally immature patients. The promising results suggest that this technique may enhance graft maturity, representing a potential advancement in the treatment of this challenging patient population.

## CONCLUSION

Over‐the‐top anterior cruciate ligament reconstruction with preserved hamstring insertion and lateral extraarticular tenodesis in skeletally immature patients results in early graft maturity, with no significant differences at 4 months compared to an adult population at either 4 months or 18 months post‐surgery. These preliminary results suggest that employing this technique could favour the ligamentization process, although future studies are needed to investigate the clinical effect of these findings.

## AUTHOR CONTRIBUTIONS

**Yuta Nakanishi**: Manuscript writing. **Alberto Grassi**: Manuscript design; surgeries; manuscript writing. **Luca Ambrosini**: Statistical analysis; manuscript writing. **Emanuele Altovino**: Manuscript writing. **Claudio Rossi**: Data collection and analysis. **Emre Anil Özbek**: Manuscript writing. **Stefano Zaffagnini**: Critical review; surgeries.

## CONFLICT OF INTEREST STATEMENT

No Disclosure: Yuta Nakanishi, Luca Ambrosini, Emanuele Altovino, and Claudio Rossi. Alberto Grassi: Smith & Nephew: Not paid consultant. Emre Anıl Özbek: Acta Orthopaedica et Traumatologica Turcica: Editorial or governing board. Smith & Nephew: Paid presenter or speaker. Turkish Society of Orthopaedics and Traumatology: Board or committee member. Stefano Zaffagnini: DePuy, A Johnson & Johnson Company: Paid presenter or speaker, paid consultant. European Society of Sports Traumatology Knee Surgery and Arthroscopy (ESSKA): Board or committee member. International Society of Arthroscopy, Knee Surgery, and Orthopaedic Sports Medicine (ISAKOS): Board or committee member. *Journal of Experimental Orthopaedics* (JEO): Editorial or governing board. Smith & Nephew: Paid presenter or speaker, paid consultant.

## ETHICS STATEMENT

Approval for this study was obtained from the local Ethical Committee (CE‐AVEC 380/2019/Oss/IOR, protocol number 0006881). Informed patient consents were signed for all patients' families because the patients included in this manuscript were under 18‐years‐old.

## Data Availability

The authors decide not to share data of this manuscript with readers or others.
